# What Works in Violence Prevention Among Young People?: A Systematic Review of Reviews

**DOI:** 10.1177/1524838020939130

**Published:** 2020-07-17

**Authors:** Anastasiia G. Kovalenko, Charles Abraham, Ella Graham-Rowe, Mark Levine, Siobhan O’Dwyer

**Affiliations:** 1 University of Exeter, United Kingdom; 2 University of Melbourne, Parkville, Australia; 3 Lancaster University, United Kingdom

**Keywords:** review of reviews, narrative reviews, meta-analyses, young people, campus violence prevention

## Abstract

Violence prevention programs aim to raise awareness, change attitudes, normative beliefs, motivation, and behavioral responses. Many programs have been developed and evaluated, and optimistic claims about effectiveness made. Yet comprehensive guidance on program design, implementation, and evaluation is limited. The aim of this study was to provide an up-to-date review of evidence on what works for whom. A systematic search of PsycINFO, MEDLINE, ERIC, and Sociology Collection ProQuest identified 40 reviews and meta-analyses reporting on the effectiveness of violence prevention programs among young people (age 15–30) in educational institutions, published before October 2018. These included reviews of programs designed to reduce (i) bullying, (ii) dating and relationship violence, (iii) sexual assault, and (iv) antisocial behavior. Only evaluations that reported on behavioral outcomes such as perpetration, victimization, and bystander behavior were included. The reviewed evaluations reported on programs that were mainly implemented in high-income countries in Europe and North America. The majority found small effects on violence reduction and victimization and increases in self-reported bystander behavior. Our findings expose critical gaps in evaluation research in this area and provide recommendations on how to optimize the effectiveness of future programs.

Many campus-based violence-prevention programs have been developed. For example, in the United States, the 2013 Campus Sexual Violence Elimination Act made campus-based sexual violence prevention programs mandatory, requiring educational institutions to report statistics on dating and sexual violence and provide support to survivors. Evaluation results have been mixed; some have demonstrated significant positive effects on knowledge and awareness, but few have measured behavior change (e.g., bullying—Stevens et al., 2000; sexual assault—Taylor et al., 2011, 2013). Of those that have measured behavior, some have shown reductions in perpetration or victimization (e.g., dating violence—Foshee et al., 2004, 2005; sexual assault—Taylor et al., 2011), but several have proved harmful (increasing perpetration and victimization rates; e.g., bullying—Roland, 1989; sexual assault—Stephens & George, 2009), and others have shown no effect (e.g., substance abuse–related violence—Newton et al., 2010). Varying designs and target populations make it challenging to determine what works to prevent each type of violence, for whom, and what is critical to optimizing effectiveness.

Systematic reviews and meta-analyses have been reported (e.g., DeGue et al., 2014—sexual assault; Polanin et al., 2012—bullying; Cox et al., 2016—substance abuse–related violence and antisocial behavior), and several reviews of reviews have accumulated the results and recommendations of these reviews (Lester et al., 2017; Matjasko et al., 2012; Zych et al., 2015). Lester and colleagues (2017), for example, synthesized findings from 36 reviews of prevention programs targeting intimate partner violence, antisocial behavior, and bullying. Their findings suggest that despite the prevalence of sexual assault rates in Africa, Eastern Mediterranean, and Southeast Asia, most programs are implemented and evaluated in the United States and that behavior, especially victimization, is rarely assessed as an outcome. Matjasko and colleagues (2012) conducted a systematic meta-review of 37 meta-analyses and 15 systematic reviews on youth violence prevention. Among those, 15 reported on school-based programs targeting bullying, antisocial behavior, and substance abuse–related violence. Eleven reviews and meta-analyses reported moderate to large effects, while four meta-analyses and reviews showed small but significant effects on youth violence–related outcomes. Zych et al. (2015) reported on 66 reviews of programs targeting bullying and cyberbullying, concluding that programs were effective in reducing perpetration and victimization, but effects were small. While these reviews provide important syntheses of the available evidence, including some evidence on the moderator effects of program features (e.g., Matjasko et al., 2012), they do not provide a clear set of evidence-based design recommendations.

## The Present Study

We conducted a systematic review of reviews of evaluated campus-based programs designed to reduce violence among young people. The objective was to synthesize the existing evidence, determine what works and why, and make recommendations on the development and implementation of future programs. Four research questions were addressed:

1. How effective are campus-based violence-prevention programs in reducing perpetration and victimization, and increased self-reported bystander helping behaviors?
2. Is effectiveness, assessed by behavior change, enhanced by specific program features?
3. Can evidence-based recommendations be made on how to improve the effectiveness of prevention programs for particular groups, depending on types of violence?
4. How can the evaluation of such programs be improved?

## Method

The conduct of this review followed a published protocol registered in PROSPERO 2019 (CRD42019109004; Kovalenko, Abraham, & Graham-Rowe, 2019).

### Literature Search

The search strategy was developed by the first and second author in consultation with experts and used a combination of relevant free-text terms (e.g., school AND (violen* OR rape OR bully* OR antisoc*) AND (program* OR reduc* OR prevent) AND (review* OR meta*)). The following electronic databases were searched from inception to October 2018: PsycINFO, PsycArticles, MEDLINE, ERIC, Sociology Collection ProQuest. In addition, the reference lists of identified reviews of reviews were searched for relevant papers. Articles were also retrieved from other sources (e.g., ResearchGate, email correspondence with researchers). For the full list of key words, see Appendix A in the Supplemental Document.

### Study Selection and Eligibility Criteria

Studies were initially selected using seven broad inclusion criteria, namely, (1) they were published in English; (2) reported a systematic review, narrative review, or meta-analysis; (3) reviewed (at least in part) experimental and quasi-experimental evaluations of (4) programs designed to reduce or prevent violence among (5) young people (6) including a sample of (at least in part) 15–30 years old and (7) set (at least in part) in educational institutions (school, college, or university). We focused on the assessment of behavior change outcomes because, while knowledge, attitudes, perceived norms, and intentions are the important precursors of behavior and legitimate indicators of psychological change, they do not necessarily predict behavior (De La Rue et al., 2014; McMahon et al., 2017).

Eligible studies reported on one or more of four categories of violence: (i) bullying, (ii) dating and relationship violence, (iii) sexual assault, and (iv) antisocial behavior. Studies that reviewed both community and educational institution-based programs were reviewed, but only findings related to educational institutions were included. Eligibility criteria were applied to all unique titles and abstracts by the first author, while the third author reviewed 10% of titles and abstracts. Full texts meeting the inclusion criteria were retrieved, and eligibility criteria applied in the same way. Any discrepancies were resolved through an email discussion.

### Study Quality Assessment

Study quality was assessed using the Measurement Tool to Assess Systematic Reviews (AMSTAR 2;Shea et al., 2017). Each review was scored against a checklist of 16 standard items representing seven critical and nine noncritical domains. Reviews with none or just one noncritical weakness were considered as high quality, reviews with more than one noncritical weakness were rated as moderate quality, and reviews with one critical flaw with or without additional noncritical weaknesses were rated as low quality. Critically low quality was indicated by more than one critical flaw. If the paper had multiple noncritical weaknesses, the overall confidence rating was moved one category down. About 10% of studies were double coded, and discrepancies were resolved with the team. The number of potential agreements and the number of actual agreements were calculated for each paper. The percentage was summed and divided by the number of papers.

### Data Extraction

Information was extracted on (1) programs included (e.g., population, outcome measures, program format) and (2) review methods, outcome measures, conclusions, and recommendations. A full data extraction table is available from the authors.

Each review was searched for descriptions of characteristics of relevant programs and for both statistical and narrative assessment of the relationship between characteristics and effectiveness by the four types of violence targeted. A narrative synthesis (Popay et al., 2006) was undertaken, and thematic analysis (Braun & Clark, 2006) was employed to summarize review recommendations in relation to program type.

## Results

**Figure 1.**
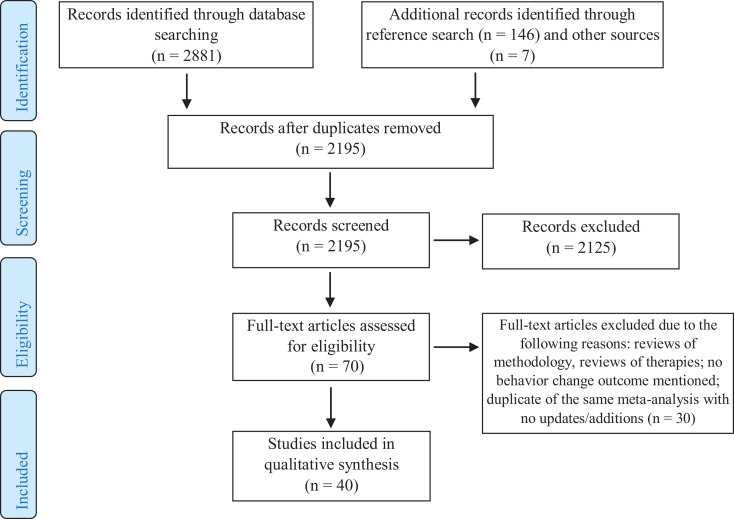
Preferred Reporting Items for Systematic Reviews and Meta-Analyzes flowchart.

This review is reported in accordance with the Preferred Reporting Items for Systematic Reviews and Meta-Analyses (PRISMA) guidelines (Moher et al., 2009). The database search identified 2,881 papers (see Figure 1); 153 additional papers were retrieved from reference lists and other sources. The reliability of inclusion selection was checked in two stages. After removing duplicates, 2,195 titles and abstracts were screened by the first author; 10% of the papers were then reviewed by the third author. The interrater agreement at this stage was 98%. After the resolution of discrepancies, 70 full-text papers were retrieved and screened by the first author. Thirty of those did not meet the inclusion criteria and were removed. At Stage 2, 14% of excluded and included full-text papers were reviewed by the third author. Interrater agreement was 95%, and discrepancies were resolved through discussion.

### Review Characteristics

Forty reviews of violence prevention programs, published between 1999 and 2018, met the inclusion criteria (and are marked with an asterisk in the reference section). Of these, 19 (47.5%) were systematic reviews and 21 (52.5%) were meta-analyses. Reviews were mainly international. The majority reported on evaluations conducted in North America (22), Australia (7), and Europe (9), while only a few—in Latin America (2), South Africa (2), and Asia (2). Sixteen reported on programs implemented in schools, four reviewed college-based programs, and two reviews included both school- and university-based programs. The rest (18) were implemented in more than one setting, including those outside campuses. Bullying and antisocial behavior prevention programs targeted populations of 5–18 years old, while rape prevention programs focused on populations 11–29 years old.

### Review Quality

Interrater agreement of 83% was achieved for study quality, and disagreements were resolved through discussion. Significant weaknesses were found in all studies. Of the 40 reviews, one was considered to be of high quality, one of medium, one of low, and, worryingly, 37 (92.5%) of critically low quality. Intervention funding sources were not reported or investigated by the reviews. Thirty-eight reviews (95%) did not include or mention a predetermined review protocol. Thirty-seven papers did not provide a list of excluded studies. Only 15 reviews adopted the Population, Intervention, Comparison and Outcome (PICO) reporting structure, and the lack of detailed information about interventions and populations prevented identification of characteristics of effective programs. Since this was, to our knowledge, the first summary of evidence-based recommendations mapping program characteristics that could work in violence prevention, we decided that inclusion of all reviews, including those of critically low quality, would be beneficial to provide a more comprehensive overview of the field. In the Supplemental Document (Appendix B), we provide study-by-study quality ratings including critical and noncritical criteria. The reader can decide which recommendations to be guided by, in view of the methodological quality of the studies supporting each recommendation.

### Violence Type Considered

Eight reviews reported exclusively on bullying, 10 targeted dating and relationship violence, 5 focused on sexual assault, and 8 included antisocial behavior–related programs (Appendix C in Supplemental Document). In addition, nine reviews considered more than one type of violence, including substance abuse–related harm and were labeled “general.”

### Behavior Outcome Measures

The most commonly reported measures were self-reported perpetration and victimization, and less often—violence prevalence rates, teacher observations, or self-reported witnessed incidents. We identified 13 reviews that included programs with a bystander component, but only eight reported bystander behavior outcomes. These targeted sexual assault (4), dating and relationship (3), and bullying (1) prevention. Overall conclusions regarding behavior change are summarized in the final column with the number of studies used to draw these conclusions.

### Review Findings

The majority of the reviews reported small posttest effects on reduction of perpetration and victimization in populations of 15 years or older (compared to baseline). With only a few exceptions, program effects decreased at follow-up. Several reviews reported mixed results with harmful or no effects for behavior change.

#### Bullying prevention

Baldry and Farrington (2007) reviewed 16 evaluations that assessed student bullying reported by teachers and student self-reported perpetration and victimization. Three evaluations produced small desirable outcomes for perpetration and victimization in students aged 15 and older (Baldry & Farrington, 2004; Olweus, 1991, 1993; Ortega & Lera, 2000), but five reported mixed results with harmful effects. One of the effective programs targeted different levels—individual, classroom, and school, and adoption of certain rules. Another taught cognitive and social skills to understand emotions of others using video, role-play, and booklets. The third effective program incorporated a positive approach to bullying prevention, implementing alternative conflict management strategies, problem-solving solutions, and peer support groups.

Ferguson et al. (2007) reviewed 45 studies, all of which produced statistically significant positive results, but the effect sizes were too small to make practical differences to institutional cultures. In addition, missing information on evaluation outcomes meant that conclusions about specific program components could not be drawn.

Lee et al. (2015) conducted a meta-analysis of 13 studies. Four targeted students up to 15 years old and produced small to moderate effects on victimization (Berry & Hunt, 2009; Houlston & Smith, 2009; Stevens et al., 2000; Wong et al., 2011). The effect sizes were larger for studies that incorporated emotional control and peer-counseling components.

In their review of 32 studies, Nocentini and colleagues (2015) included nine relevant programs, including online and off-line components. Two of these studies demonstrated significant reductions in bullying perpetration and victimization (Karna et al., 2013; Palladino et al., 2016); the latter remained effective at a 6-month follow-up for adolescents compared with the control group. This was a universal peer-led program in a school context with an online and off-line component. The other program, evaluated by Karna et al. (2013), was aimed at raising awareness about bystander intervention and building empathy toward victims.

Polanin et al. (2012) reviewed 11 evaluations of bystander-intervention programs. Their meta-analysis indicated that program participants intervened significantly more often to prevent bullying, compared with controls, but perpetration or victimization was not reported. High school samples showed significant small to moderate effects. No differences were found between United States and European samples, or in the length of programs (1–2, or 6–12 months).

In their review of 14 studies, Smith et al. (2004) included five programs targeting students aged 15 years and older. Three evaluations reported positive (but very small) effects. Programs incorporated school policies and classroom rules, increased supervision, and targeted interventions for bullies and victims. Two evaluations produced mixed/negative results for perpetration and victimization.

Ttofi and Farrington (2011) reviewed 89 studies and conducted a meta-analysis of 44 programs. School-based programs were effective in reducing perpetration and victimization. More intensive, longer programs (of 20 hr and over 270 days) were more effective, while, worryingly, engagement of peers in peer mentoring or mediation increased victimization. Disciplinary methods (e.g., firm sanctions, serious talks with bullies, depriving of privileges), videos, and group work were significantly related to reductions in perpetration and victimization.

In a systematic review of 26 programs, Vreeman and Carroll (2007) included 10 evaluations of whole-school programs, including four implemented in secondary schools. Only one evaluation reported significant reductions at follow-up (Olweus, 1994); however, it was not possible to determine outcomes separately for students aged 15 and over.

#### Dating and relationship violence prevention

De Koker et al. (2014) reported on six programs, among which three had positive effects on reduction of psychological and physical perpetration (Foshee et al., 2005; Taylor et al., 2011; Wolfe et al., 2009), were activity-based, and focused on key adult mentors (e.g., teachers, parents, and community members). The first 10-session program focused on feminist and social learning theory, included a peer theater production, a poster contest, community activities, and support services for adolescents experiencing relationship violence. This gender-neutral program was equally effective for both genders. The second, 21-session program, involved skills-based learning in gender-segregated classes focusing on personal safety, health and sexuality, sexual decision making, dealing with pressure, and problem-solving. The third program was based on the theory of reasoned action and comprised six sessions including legislation and consequences, construction of gender roles, healthy relationships, and the role of bystanders.

Edwards and Hinsz (2014) reviewed eight studies that produced a small effect on dating violence–related outcomes, including behavior. Despite sample sizes being too small for moderation analyses, the authors concluded that school-based programs were more effective for younger students as opposed to teenagers.

Leen et al. (2013) reviewed nine evaluations of dating violence programs. Four of these focused on behavior change outcomes and two found a positive effect on behavior at posttest and follow-up (Foshee et al., 2004; Wolfe et al., 2009). However, surprisingly, participants receiving additional (booster) sessions in the intervention reported by Foshee et al. (2004) reported increased victimization compared to those who did not receive these sessions. The review suggested that, in comparison with programs aimed at improving knowledge and attitudes, programs that targeted behavior change were more effective at follow-up (6 months to 4 years).

Malhotra et al. (2015), in their review of 18 programs, included 12 that were school-based, implemented in students aged 15 and over, with mixed effects. The programs reduced perpetration and victimization, but the effects of all but one program (Foshee et al., 1998, 2000, 2004, 2005—mentioned earlier) were not maintained at follow-up, and one reported increased perpetration.

In their review of 14 studies, Petering et al. (2014) included seven programs targeting youth in school settings. Positive effects for behavior (perpetration, victimization, or bystander helping) were found in three evaluations of programs implemented schoolwide among students aged between 14 and 18 years old in comparison with controls (Foshee et al., 2005; Miller et al., 2012; Wolfe et al., 2009). Effective programs were intensive, lasting between ten 45-min and twenty-one 1-hr sessions. These programs included poster sessions and theater performances and facilitation by male athletic coaches.

Storer et al. (2016) reviewed nine bystander programs evaluated across 15 studies. Four studies produced a small positive effect (Barone et al., 2007; Coker et al., 2011; Foubert et al., 2009; Moynihan et al., 2015), three had mixed effect on bystander behavior, and two other programs produced no significant effect.

Whitaker et al. (2006) reported on 11 programs, of which 1 reported decreases in physical perpetration, where the effect was larger for girls, but with no effects for victimization (Wolfe et al., 2003). This program utilized a health promotion approach and feminist theories regarding societal values. Activities included presentations, videos, role-playing, and skill-building activities. Another program that produced small effects on perpetration and victimization was mentioned in other reviews (Foshee et al., 1998).

In addition, Whitaker et al. (2013) reviewed 19 studies, but all evaluations of school-based programs that produced significant results were included in other reviews mentioned above. The meta-analysis of 23 studies conducted by De La Rue et al. (2017), and the review of 38 studies by Fellmeth et al. (2013) found no statistically significant improvement in postintervention behavior compared with controls.

#### Sexual assault prevention

Anderson and Whiston (2005) reviewed 102 programs focusing on rape knowledge, attitudes, and self-reported behaviors in sexual assault contexts. Programs showed practically negligible effects on behavior. Effective programs were presented by trained professionals and included content addressing risk factors, gender roles, and/or myths and facts about sexual assault. There was support for mixed- and single-gender sessions, but results suggest that single-gender delivery is more effective in strengthening women’s behavioral intentions. Focused programs targeting only a few topics in-depth were more effective in improving behavior than programs with multiple topics.

DeGue et al. (2014) reviewed 140 studies, among which 84 were single-session programs in college settings lasting for 68 min on average. Only two (2.4%) of these programs were effective in the long term for behavioral outcomes such as perpetration, victimization, and violence prevalence rates (Boba & Lilley, 2009; Foshee et al., 1998, 2000, 2004, 2005). The majority of evaluations reported significant positive effects on knowledge and attitudes. The reviewers concluded that programs lasting 6 hr or more were more effective and suggested that programs be developed in accordance with effective prevention strategies such as Nation et al.’s (2003) “nine principles of prevention.”

Jouriles et al. (2018) conducted a meta-analysis of 24 bystander programs. Although there was a small but significant improvement in interventions to prevent rape posttest, this decreased at follow-up. Length of programs, type of facilitator (peer or nonpeer), and mode of delivery (e.g., video, online, or poster campaigns) were not associated with effectiveness in relation to behavior change.

Katz and Moore (2013) reviewed 12 evaluations of campus-based bystander programs. Overall, moderate effects were found for intent to help and bystander efficacy, along with small but significant improvements in bystander helping behaviors, for example, verbally disapproving of a sexist comment or joke. There was no effect on perpetration compared with baseline or postintervention controls. Studies with a higher proportion of male participants showed larger significant effects on intent to help, and the reviewers suggested that younger students might feel more empowered by such programs.

Kettrey and Marx (2019) reviewed 15 bystander programs. The effects on bystander helping behavior were small but significant compared with controls, and the reviewers found greater improvement in bystander efficacy and intent to help students in early college years.

#### Antisocial behavior prevention

Derzon (2006) reported on 83 programs targeting antisocial behavior in school settings. Among those, 13 evaluations reported significant reductions in physical violence and student fighting. It was unclear, however, what age groups were targeted, and what promoted effectiveness.

Fields and McNamara (2003) compared primary and secondary intervention effects. The reviewers reported that, while the 16 primary programs, aiming to prevent an incident, showed small effects on various outcomes including behavior change, secondary programs, providing response to at-risk populations after an incident happened, were more effective. Several programs found decreases in violent behaviors when the intervention was short term with a social learning component, delivered in schools; however, the age of populations was unclear.

Gavine et al. (2016) reported on 21 evaluations of 16 programs. Several reported significant reductions in perpetration and nonphysical aggression in populations aged 15 and over compared with controls (Castillo et al., 2013; Yeager et al., 2013). Effective programs combined social norms promotion and developmental approaches with a problem-solving and decision-making skill-building component, a peaceful-conflict management training. A lack of long-term follow-up did not allow sustainability of effects to be assessed.

Lösel and Beelmann (2003) found small but significant positive effects overall across 84 evaluations compared with controls. Programs that targeted at-risk populations reported greater improvements compared to general school-based programs.

Park-Higgerson et al. (2008) reviewed 26 school-based studies that produced no significant difference in effects for behavior between intervention and controls. Perhaps surprisingly, multiple-approach programs that involved peers, families, or communities did not show any evidence of benefit while single-approach programs demonstrated significant positive effects.

Sawyer et al. (2015) reviewed 66 interventions in their meta-analysis. Overall reductions in antisocial behavior were small at posttest compared with controls. It was unclear, however, which characteristics enhanced effectiveness in students aged 15 and older.

Wilson et al. (2003) included 221 studies, and this was updated by Wilson and Lipsey (2007) to include 249 evaluations of school-based programs. A small, significant decrease in aggressive behavior at posttest was observed across all age groups with no difference by gender. Younger students, those from lower low socioeconomic status, showed larger effects. Whole-school programs including cognitive components were most effective. Larger effects were achieved in populations with a higher prevalence of violence. Comprehensive, multicomponent schoolwide interventions involving a mix of various formats across settings, such as social skills building and parental training, were surprisingly ineffective.

#### General violence prevention

In their review of 10 programs, Atienzo et al. (2017) included one *bullying prevention program* that included populations 15 years and older and reported on behavioral outcomes. The study reported significant reductions in witnessed bullying (Perez et al., 2013). The main activities included skills development in classroom; individual meetings with students, teachers, and parents; bullying detection and monitoring; and a bullying complaint mailbox. In addition, the review included two evaluations of programs targeting *antisocial behavior* that reported behavioral outcomes in the middle school student population, including ages 15–16. Small but significant effects were found for reduction of perpetration and witnessed violence in one study that involved activities such as skills development in classroom, improvement of physical environment in school, individual counseling to students, and meetings with parents and community (Varela, 2011). Another program reported increased involvement in deviant activities and included modification of school rules and training in mediation (Kenney & Godson, 2002).

In their review of 19 evaluations of 17 general youth violence prevention programs, Cox et al. (2016) included 1 *bullying prevention* and 1 *antisocial behavior prevention program* with mixed results for student populations aged 15 and older. The bullying prevention program reduced perpetration, however, the sample was small (*N* = 25; Hunt, 2007). The school-based antisocial behavior training program focused on positive conflict management skills and was found to generate a significant decrease in violent behaviors compared with controls (Bretherton et al., 1993). Overall, the authors concluded that whole-school, comprehensive programs across the types of violence were the most effective compared with targeted and social skills programs.

Fagan and Catalano (2013) reviewed 17 studies, among which 1 evaluated a program targeting *dating violence* in high schools and 1 reviewed a media-based middle school *bullying prevention* program that included populations aged 15 at follow-up. Small to medium effects were found for violence reduction in these studies (Foshee et al., 1998, 2005; Swaim & Kelly, 2008) including long-term effects for up to 3 years. The dating violence prevention program was included in other reviews. The media-based bullying prevention curriculum was aimed at increasing respect for individual differences and promoting conflict resolution. High school students designed print, TV, and radio media and organized events to promote violence reduction.

Hahn et al. (2007) reviewed 53 school-based violence prevention programs, noting that the focus shifted from behavior modification for general antisocial behavior in elementary and middle schools to skills training for specific types of violence in middle and high schools. Four universal school-based programs produced small effect for violence reduction in high school sample (Foshee et al., 2000; Kenney & Watson, 1996; Renfro et al. 2003; Shapiro et al., 2002). Length of the program was not associated with effectiveness. All school program strategies proved to be effective (e.g., informational, social skills building, student, peer, other facilitator status except for school administrators).

Howard et al. (1999) reviewed 44 evaluations of school-based programs, among which 1 targeted *antisocial behaviors* among students 15 years and older. This program reported reductions in suspension rates compared to baseline (Hansman et al., 1996). Curriculum focused on knowledge about risk factors, nonviolent ways to respond, and anger management.

In their meta-analysis of 14 programs, Jiménez-Barbero et al. (2016) reported on two programs targeting students aged 15 or older. An anti-bullying program reduced victimization in older students but increased perpetration and victimization in younger populations (Baldry & Farrington, 2004). The antisocial behavior program did not produce significant results.

Three reviews conducted by Cassidy et al. (2016), Limbos et al. (2007) and Scheckner et al. (2002) reported no effects on behavior in populations of interest. Nevertheless, Cassidy et al. (2016) suggested that multilevel media campaigns might be effective. A few reviews reported on programs designed to reduce substance abuse–related violence (Cox et al., 2016; Fagan & Catalano, 2013; Hahn et al., 2007; Jiménez-Barbero et al., 2016). Evidence of effectiveness was very limited. The reviews suggested that, as with other interventions, programs should be tailored to account for cultural and social differences (Hahn et al., 2007) and include skills training (e.g., negotiation, conflict resolution, and peer support—Cox et al., 2016).

### Overall Recommendations

The majority of reviews provided recommendations on how to improve program effectiveness. Thematic analysis of the recommendations revealed several repeating themes in multiple reviews, and these are reported in relation to program type. Reviews also provided guidelines on robust evaluation. These are summarized across program types (see Table 1).

**Table 1.** Program Recommendations.

Characteristics Likely to Enhance Effectiveness	Bullying	Dating and Relationship	Sexual Assault	Antisocial
Curricula that are longer and more intensive	At least 20 hr delivered over longer periods (e.g., Ttofi & Farrington, 2011)	*Effective programs had a 10- or 21-session curriculum, each session lasting between 45 and 60 min (De Koker et al.,* *2014* *)*	Comprehensive long-lasting programs incorporating multiple sessions (Anderson & Whiston, 2005; DeGue et al., 2014; Jouriles et al., 2018; Katz & Moore, 2013).	*Short-term programs with a social learning component, as well as programs targeting at-risk populations, were effective (Fields & McNamara,* *2003* *; Losel & Beelmann, 2* *003)*
Comprehensive curricula including multiple change strategies and a variety of delivery modes	A whole-school well-planned approach that involves multiple disciplines (e.g., a combination of school rules and sanctions, classroom and individual training, teacher training) (Vreeman & Carroll, 2007). Multiple modes, including media (e.g., video, booklet), face-to-face interaction, and physical environment redesign (Howard et al., 1999; Vreeman & Carroll, 2007). Involvement of families (Ttofi & Farrington, 2011). Implementation of school policies promoting bullying prevention and intervention (Lee et al., 2015).	Prevention should be incorporated into school policies (De La Rue et al., 2014). Multiple modes, e.g., poster, video, role-play (De Koker et al., 2014)	*Effective programs addressed healthy relationship skills, gender roles, risk factors, and social norms (Anderson & Whiston,* *2005* *; DeGue et al.,* *2014* *).* *Effective programs included theater play and poster contest (DeGue et al.,* *2014* *).*	Multi-component initiatives that combine several approaches, including social norms (Gavine et al., 2016), risk (e.g., substance abuse, school dropout—Fields & McNamara, 2003) and protective factors (Fagan & Catalano, 2013) were effective. *Effective programs involved individual counseling, meeting with parents, and improvement of the physical environment at school (Atienzo et al., 2017)*
Curricula employing change strategies derived from evidence-based theories	Bullying perpetration and victimization (Baldry & Farrington, 2007); Social norms and social identity approach: Bullying behaviors should be regarded as a group process where each participant has their role and social status and treated accordingly (Polanin et al., 2012).	Developmental theory (Storer et al., 2016) Feminist theory, social learning theory (De Koker et al., 2014) Background-situational theory, coercive interactional processes, health promotion and development of healthy relationships (Whitaker et al., 2006)		*Effective programs targeted social norms and developmental approach (Gavine et al.,* *2016* *)*
Curricula including specific skills training	Training on emotional control, peer counseling (Lee et al., 2015), empathy (Polanin et al., 2012; Ttofi & Farrington, 2011); problem-solving solutions, and peer support. Social and perspective-taking skills components (Ttofi & Farrington, 2011; Vreeman & Carroll, 2007). Playground supervision component, “hot spots” (Ttofi & Farrington, 2011).	Incorporate skill-building content and opportunity to practice (e.g., conflict resolution, problem-solving, sexual decision making, dealing with pressure—DeKoker et al., 2014; De La Rue et al., 2014)	Recommended integrating more active learning approaches focusing on behavioral involvement, bystander education, the role of relationships, and risk factors (DeGue et al., 2014; Katz & Moore, 2013)	Skill development components (e.g., negotiation skills, conflict resolution, and peer support—Cox et al., 2016) *Effective programs included problem-solving and peaceful conflict management training (Gavine et al., 2016)*
Programs targeting behavior change (rather than belief change) and evaluated in terms of behavior change.	Behavior change (perpetration and victimization) should be explicitly targeted and assessed in evaluations (Polanin et al., 2012). In addition to self-reports, observer/teacher reports, and systematic observation should be employed to strengthen evidence (Baldry & Farrington, 2007).	Focus on physical and psychological perpetration, emotional and verbal abuse, victimization, prevalence rates, and bystander helping behaviors as the target outcome measure (Leen et al., 2013; Malhotra et al., 2015; Whitaker et al., 2006).		Effective programs should ensure behavior change (Cassidy et al., 2016)
Curricula tailored to the target population	Special attention should be drawn to student needs and school climate assessment (Polanin et al., 2012).	Programs should be appropriately tailored to the culture and needs of target audiences (Fellmeth et al., 2013; Whitaker et al., 2006)		Assessment of student needs to help, develop, and refine effective curricula and ensure sustained change along with process evaluation (Gavine et al., 2016). Specific population-targeted in-depth programs could be more efficient than universal programs (Cassidy & Matzopoulos, 2016; Gavine et al., 2016).
Curricula tailored to the target population	Special attention should be drawn to student needs and school climate assessment (Polanin et al., 2012).	Programs should be appropriately tailored to the culture and needs of target audiences (Fellmeth et al., 2013; Whitaker et al., 2006).		Assessment of student needs to help, develop, and refine effective curricula and ensure sustained change with process evaluation (Gavine et al., 2016). Specific population-targeted in-depth programs could be more efficient than universal programs (Cassidy & Matzopoulos, 2016; Gavine et al., 2016).
Curricula that incorporate cultural diversity and careful implementation in different cultures/countries		Further programs are needed outside North America and in low- and middle-income countries (Fellmeth et al., 2013; Leen et al., 2013; Malhotra et al., 2015)	Programs should be geared toward multiple populations to ensure the diversity of samples (DeGue et al., 2014).	The impact of sociodemographic characteristics should be taken into account (Atienzo et al., 2017). Outcome and process evaluation should be done beyond North America, in low- and middle- income countries (Gavine et al., 2016).
Curricula that take account of different gender needs		Programs should include a gender-specific approach or be gender-neutral (De Koker et al., 2014), where curricula should incorporate various scenarios whereby men are not always perpetrators and women not always victims.	Targeting both single- and mixed-gender groups could maximize the effectiveness (Anderson & Whiston, 2005; DeGue et al., 2014; Jouriles et al., 2018; Katz & Moore, 2013). Bystander programs facilitated or attended by men could show a positive example of masculinity and helping (Katz & Moore, 2013).	Programs should be taking into account gender differences (Atienzo et al., 2017)
Appropriately timed programs	Ferguson et al. (2007) stated that a lack of effectiveness might have been due to poor timing such as implementation after school bullying had already decreased naturally		Programs could be implemented with younger students (Katz & Moore, 2013; Kettrey & Marx, 2019) or adolescents (DeGue et al., 2014) and assessed throughout the college years.	
Programs evaluated using quality, experimental designs	Independent, robust experimental evaluations (Baldry & Farrington, 2007; Jiménez-Barbero et al., 2015)	Large, longitudinal, well-conducted RCTs would provide more definitive evidence of effectiveness (Fields & McNamara, 2003; Katz & Moore, 2013; Malhotra et al., 2015; Petering et al., 2014; Storer et al., 2016). Use reliable validated scales to measure outcomes (Fellmeth et al., 2013)		Evaluations of antisocial and aggressive behavior prevention programs should have a random allocation to intervention and control groups (Fields & McNamara, 2003)
Program evaluations including planned sub-group or moderator analyses		Investigate relationships between the outcome measures, including attitudes, knowledge, behavior, and actual cases of violence using mediational analyses (Fellmeth et al., 2013). Moderation analysis to identify who is most likely to change and when programs are best implemented (Storer et al., 2016).The effect of increased bystander behavior on decreases in violence rates should be explored (Katz & Moore, 2013; Polanin et al., 2012).	Further meta-analytic investigations are needed to assess the effect of specific program features, content components, delivery methods on outcome measures, as well as the effectiveness of delivery by peer versus professional staff (DeGue et al., 2014; Katz & Moore, 2013)	Study the effect of specific program components on outcome measures and populations (Atienzo et al., 2017; Cassidy et al., 2016; Fagan & Catalano, 2013)
Miscellaneous recommendations	Develop a system of accrediting anti-bullying programs to enable replicability with fidelity of the most effective interventions (Ttofi & Farrington, 2011). Perpetration and victimization should be measured at least twice per month (Ttofi & Farrington, 2011) and cost-effectiveness calculated.	Terms such as aggression, rape, and dating violence should be clearly defined (Edwards & Hinsz, 2014; Fellmeth et al., 2013)	Clarify the utility of booster sessions and provide better maintenance data by using longer follow-up times (Katz & Moore, 2013; Kettrey & Marx, 2019).	The most effective approaches should be integrated into nationally funded programs (Fields & McNamara, 2003). Training students as group facilitators to diffuse new norms and ensure cost-effectiveness (Jouriles et al., 2018).
Programs reported in detail to allow accurate replication	Detailed reports and data should be made available to other researchers and designers to enable reanalyses and evidence-based intervention development (Ttofi & Farrington, 2011)	Evaluations should report data consistently (Edwards & Hinsz, 2014)		Effect sizes and clinical significance should be reported to allow more meaningful interpretation (Fields & McNamara, 2003) Further replication is required and to allow this, consistent reporting is needed (Fagan & Catalano, 2013).

*Note*. RCTs = randomized controlled trials. Recommendations are written in regular font, while conclusions about effective programs—in italic.

#### Bullying prevention

Reviews suggested that anti-bullying programs should be well planned (Vreeman & Carroll, 2007), intensive, and of longer duration (Ttofi & Farrington, 2011). Curricula should be based on (i) theories of bullying perpetration and victimization (Baldry & Farrington, 2007) and include training in (ii) empathy (Polanin et al., 2012; Ttofi & Farrington, 2011), social perspective-taking (Ttofi & Farrington, 2011; Vreeman & Carroll, 2007), (iii) emotional control, (iv) problem-solving, and (v) peer counseling. Whole-school approaches involving school rules and sanctions should be used to prompt student and teacher training. Howard et al. (1999) argued that programs should use multiple delivery modes, including media (e.g., video), face-to-face interaction, and physical-environment redesign and ensure consistency and complementarity across modes. Ttofi and Farrington (2011) suggested that families should be involved in planning and implementation. Student needs, school climate (Polanin et al., 2012), and playground supervision (e.g., identification of “hot spots,” Ttofi & Farrington, 2011) should be considered. Bullying behaviors should be regarded as group processes where each participant has their role and social status and treated accordingly (Polanin et al., 2012). Ttofi and Farrington (2011) suggested that secondary school programs could be more effective because of decreasing impulsiveness and increasing rational decision making. Thus, age-tailored programs are needed.

#### Dating and relationship violence

Reviewers concluded that program content should be underpinned by evidence-based theories and appropriately tailored to the culture and needs of target audiences (Fellmeth et al., 2013; Whitaker et al., 2006). Effective programs involved peer education, use of drama and poster activities as well as education on legislation, personal safety, consequences, health and sexuality, gender roles, healthy relationships, and the role of bystanders. Dating and relationship violence prevention programs should focus on conflict resolution, problem-solving, sexual decision making, and dealing with pressure and, as with bullying programs, be incorporated into school policies (De Koker et al., 2014; De La Rue et al., 2014). They should clearly define terms such as aggression, rape, and dating violence and be gender-specific or gender-neutral (De Koker et al., 2014).

#### Sexual assault

As in other areas, reviewers suggested comprehensive long-lasting programs (e.g., at least 6 hr, DeGue et al., 2014) incorporating multiple sessions targeting both single- and mixed-gender groups could maximize effectiveness (Anderson & Whiston, 2005; DeGue et al., 2014; Jouriles et al., 2018; Katz & Moore, 2013). Katz and Moore (2013) suggested that bystander sexual violence prevention programs facilitated or attended by men could show a positive example of masculinity and helping behavior, and DeGue et al. (2014) emphasized the importance of tailoring to reach diverse targets. Active learning approaches focusing on behavioral involvement, bystander education, the role of relationships, and risk factors using drama were recommended (DeGue et al., 2014; Katz & Moore, 2013). Jouriles et al. (2018) suggested that training students as facilitators in naturally occurring peer groups could accelerate the diffusion of new group norms and could be efficient in terms of time and cost. Reviewers highlighted that sexual assault programs may be more effective if implemented with younger students (Katz & Moore, 2013; Kettrey & Marx, 2019) or adolescents (DeGue et al., 2014) and assessed throughout the college years.

#### Antisocial behavior

Reviews have shown that effective multicomponent programs combined several approaches including social norms (Gavine et al., 2016), risk (e.g., substance abuse, school dropout—Fields & McNamara, 2003), and protective factors (Fagan & Catalano, 2013) with skill development components (Cox et al., 2016) taking into account gender differences (Atienzo et al., 2017). Tailored population-targeted in-depth programs could be more effective than universal programs (Cassidy et al., 2016; Gavine et al., 2016).

#### Evaluation methodology recommendations

Reviewers have called for independent, robust experimental evaluations (Baldry & Farrington, 2007; Jiménez-Barbero et al., 2016) especially large, longitudinal, randomized controlled trials to provide definitive evidence of effectiveness (Fields & McNamara, 2003; Katz & Moore, 2013; Malhotra et al., 2015; Petering et al., 2014; Storer et al., 2016). They also call for detailed data provision that enables reanalyses and data syntheses (Ttofi & Farrington, 2011). Outcome measures should include a validated measure of behavior change, for example, of perpetration and victimization (Cassidy et al., 2016; Polanin et al., 2012; Ttofi & Farrington, 2011), emotional and verbal abuse (Whitaker et al., 2006), violence prevalence rates, and bystander helping behaviors (Leen et al., 2013; Malhotra et al., 2015; Whitaker et al., 2006). Effect sizes and clinical significance should be reported to allow a more meaningful interpretation of outcomes (Fields & McNamara, 2003). Observer and teacher reports should be used to validate self-reports (Baldry & Farrington, 2007).

Modifiable antecedents of behavior (e.g., information, beliefs, attitudes, perceived norms, and motivation (Fisher & Fisher, 1992) should be assessed using validated scales and mediation analyses conducted (Fellmeth et al., 2013). For example, does bystander empowerment decrease violence (Katz & Moore, 2013; Polanin et al., 2012)? Such process evaluations can elucidate change mechanisms and so identify modifiable targets for future programs (Moore et al., 2014). Conducting planned moderation analyses on adequately powered datasets can help identify *who* is most likely to change (e.g., in relation to age, gender, and sociodemographic status (Atienzo et al., 2017; Hahn et al., 2007) and *when* programs are best implemented (Storer et al., 2016). Further meta-analytic investigations are needed to assess the effect of specific program characteristics including curricula content (Atienzo et al., 2017; Cassidy et al., 2016; Fagan & Catalano, 2013), delivery methods (e.g., peer vs. professional staff, DeGue et al., 2014; Katz & Moore, 2013), and the use of booster sessions and to promote maintenance over time (Hahn et al., 2007; Katz & Moore, 2013; Kettrey & Marx, 2019). Better reporting is needed to allow program replication with fidelity (Fagan & Catalano, 2013), and replications and evaluations outside North America and in low- and middle-income countries are needed (Gavine et al., 2016).

## Discussion

Three decades of evaluation have generated little evidence identifying which curricula are most effective in reducing campus-based violence. The present review of reviews systematically presents this evidence and consequent recommendations for improved intervention and evaluation to answer the question “what works for whom.” We systematically reviewed 40 relevant reviews, 8 of which were published since Lester et al. (2017) reported their systematic review of reviews. With the addition of new studies, this review provides a comprehensive summary of what we know about program effectiveness across four types of violence.

### How Effective Are Campus-Based Violence Prevention Programs?

Reviews and meta-analyses demonstrated significant improvements in knowledge and attitudes. Not all programs evaluated behavior as an outcome measure, and the findings for behavior change were mixed. Small but significant reductions in perpetration and victimization were reported, but effects tended to fade or disappear at follow-up.

### Is Effectiveness Enhanced by Specific Program Features?

It is unclear how the effectiveness of programs is enhanced by specific program content. For instance, some reviews demonstrated that longer comprehensive programs were more effective, while others showed that shorter targeted programs produced stronger effects. Similarly, some programs were effective in reduction of bullying behaviors in older student populations, other programs were harmful to this age-group but were effective for younger students. Yet for each of the four violence categories, there was at least one program that produced positive effects. Based on these findings, we have summarized recommendations for each specific violence category.

### Evidence-Based Recommendations

Nation et al. (2003) suggested that programs should (a) be comprehensive, (b) be appropriately timed, (c) utilize varied teaching methods, (d) have sufficient dosage, (e) be administered by well-trained staff, (f) provide opportunities for positive relationships, (g) be socioculturally relevant, and (h) theory-driven. We have added several recommendations.

Successful campus-based violence prevention programs should include behavior change in its various forms such as physical, verbal, and emotional, as the target outcome (this would include self-reported perpetration and victimization, prevalence rates, teacher reports, and, where applicable, bystander helping behaviors). It is recommended that future programs incorporate a gender-neutral approach and conduct programs in various settings to ensure the inclusivity and diversity of populations. Program curricula should be intensive and activity-based (e.g., include peer theater play, poster contest) with a skill-building component (for instance, problem-solving, dealing with pressure, healthy relationship building). A needs assessment should be performed before development to ensure the timely and appropriate implementation (Abraham & Denford, 2020) and postintervention booster sessions considered to enhance sustained effects.

## Limitations

This review has several limitations that should be considered when implementing its recommendations. First, the lack of any unified methodological framework in the reviewed reviews presents challenges when synthesizing data and recommendations. Reporting in accordance with standardized tools using standardized behavioral outcome measures is essential for effective data analysis and presentation (Higgins et al., 2019). Second, inconsistency in reporting by type of violence in the reviews made it challenging to draw conclusions about the effectiveness by type of violence. The majority of reviews targeted bullying prevention, dating relationship violence, and sexual assault programs, and many targeted multiple violence types at once. Thus, effectiveness data may not be generalizable across program types. Third, while there was overlap in reported programs across reviews, we were unable to determine the extent of duplication. Therefore, the size of the effectiveness database is somewhat unclear. Fourth, the results should be interpreted with caution because the review included low-quality papers. Our critical appraisal revealed that the majority of these reviews did not provide adequate detail about methodology and programs included. Several reviews failed to report risk of bias in individual studies or did not adopt the PICO strategy to report the outcomes, which limited data synthesis. Fifth, we only included studies published in English so we may have missed some reviews. Sixth, the majority of programs reviewed here were evaluated in high-income countries; hence, the conclusions drawn may and have limited cross-cultural applicability.

## Conclusions

Programs proved to be effective for the improvement of knowledge and attitudes, less often for behavior, and the effect decreased over time. The lack of rigorous longitudinal evaluation design and moderator analyses limited our ability to draw conclusions about specific program features that enhance the effectiveness of violence prevention programs. Nonetheless, we provide a set of recommendations identifying best bet content and guidelines on how to improve evaluation methodology in this field.

### Implications for Research, Policy, and Practice

Despite these limitations, this review synthesizes findings from over three decades. If future programs incorporate our evidence-based recommendations, they may tackle violence among young people more effectively. Moreover, we clearly highlight the need for a more rigorous evaluation of such programs and explain how this can be achieved. When more persuasive effectiveness data are generated, the most effective approaches can be integrated into nationally funded programs embedded in everyday education practice (Fields & McNamara, 2003).

## Supplemental Material

Supplemental Material, 0306_Supplementary_Material_Online - What Works in Violence Prevention Among Young People?: A Systematic Review of Reviews Click here for additional data file. Supplemental Material, 0306_Supplementary_Material_Online for What Works in Violence Prevention Among Young People?: A Systematic Review of Reviews by Anastasiia G. Kovalenko, Charles Abraham, Ella Graham-Rowe, Mark Levine and Siobhan O’Dwyer in Trauma, Violence, & Abuse

Supplemental Material, 2602_Key_findings_and_implications - What Works in Violence Prevention Among Young People?: A Systematic Review of Reviews Click here for additional data file. Supplemental Material, 2602_Key_findings_and_implications for What Works in Violence Prevention Among Young People?: A Systematic Review of Reviews by Anastasiia G. Kovalenko, Charles Abraham, Ella Graham-Rowe, Mark Levine and Siobhan O’Dwyer in Trauma, Violence, & Abuse
