# Supplementary material for: What Works in Violence Prevention Among Young People?: A Systematic Review of Reviews
Source: Trauma Violence Abuse. 2020 Jul 17;23(5):1388–404. doi: 10.1177/1524838020939130 (PMC9606003; doi:10.1177/1524838020939130)
Supplement: Supplemental Material, 0306_Supplementary_Material_Online - What Works in Violence Prevention Among Young People?: A Systematic Review of Reviews [file 0306_Supplementary_Material_Online.pdf]

Appendix A. Search strategy.

PsycInfo and PsycArticles

((aggress* or violen* or sexual assault or bully* or anti* behav* or abus* or relationship violence or dating violence) and (intervention or program* or training or reduc* or prevent* or campaign)).ti,ab. and (review* or meta* or analysis or meta-analysis or evaluat*).ti. and (school or campus or university or college or youth or young or adolesc* or teen*).ti,ab.

Sociology collection ProQuest

ab(aggress* or violen* or sexual assault or bully* or anti* behav* or abus* or relationship violence or dating violence) and ab(intervention or program* or training or reduc* or prevention or campaign) and ti(review* or meta* or analysis or meta-analysis or evaluat*) and ab(school or campus or college or university or youth or young or adolesc* or teen*)

Medline

((aggress* or violen* or sexual assault or bully* or anti* behav* or abus* or relationship violence or dating violence) and (intervention or program* or training or reduc* or prevent* or campaign)).ti,ab. and (review* or meta* or analysis or meta-analysis or evaluat*).ti. and (school or campus or university or college or youth or young or adolesc* or teen*).ti,ab.

ERIC

(AB aggress* OR AB violen* OR AB bully* OR AB assault OR AB abus* or AB anti* behav*) AND (AB program* OR AB intervention OR AB campaign OR AB prevent* OR AB reduc* OR AB training) AND (AB school OR AB young OR AB campus OR AB teen* OR AB university OR AB college) AND (TI review OR TI meta* OR TI meta-analysis OR TI evaluat* OR TI analysis)

Appendix B. AMSTAR – 2 Quality appraisal results

	AMSTAR 2 Criteria*	Anderson & Whiston (2005)	Atienzo, Baxter, & Kaltenthaler (2017)	Baldry & Farrington (2007)	Cassidy, Bowman, McGrath, & Matzopoulos (2016)	Cox et al. (2016)	DeKoker et al. (2013)	DeLaRue, Polanin, Espelage, & Piggott (2017)	DeGue et al. (2014)	Derzon (2006)	Edwards and Hinsz (2014)
1	Population, Intervention, Comparison and Outcome	Yes	Yes	No	Yes	Uncl	Yes	Yes	Yes	Yes	Yes
2	Pre-registered protocol	No	Partial Yes	No	No	No	No	Yes	No	No	No
3	Study design criteria	Yes	Yes	No	No	No	No	Yes	Yes	No	No
4	Comprehensive literature search	No	Partial Yes	No	Partial Yes	No	Partial Yes	Partial Yes	Partial Yes	No	No
5	Study selection in duplicate	No	Yes	No	Yes	Uncl	Yes	Yes	Yes	No	No
6	Data extraction in duplicate	Yes	Yes	No	Yes	Uncl	Yes	Yes	Yes	No	No
7	List of excluded studies	No	No	No	Yes	No	No	Yes	No	No	No
8	Detail of included studies	No	Partial Yes	Partial Yes	No	Yes	Partial Yes	Partial Yes	No	No	No
9	Risk of Bias (RoB) in individual studies	Uncl	Yes	No	Uncl	Uncl	Partial Yes	Partial Yes	No	No	No
10	Sources of funding	Uncl	No	No	No	No	No	Yes	No	No	No
11	Meta-analysis – appropriate statistical methods	No	Not performed	Not performed	Not performed	Not performed	Not performed	Yes	Not performed	No	Yes
12	Meta-analysis – assess RoB	No	Not performed	Not performed	Not performed	Not performed	Not performed	Yes	Not performed	No	No
13	Meta-analysis – incorporate RoB	No	Yes	No	No	No	Yes	Yes	No	No	No
14	Explanation for any heterogeneity observed	Yes	No	No	No	No	No	No	No	No	Yes
15	Publication bias	No	Not performed	Not performed	Not performed	Not performed	Not performed	Yes	Not performed	No	No
16	Conflicts of interest reported	No	Yes	No	Yes	No	No	Yes	No	No	Yes

* Critical points are highlighted with yellow

Appendix B. AMSTAR – 2 Quality appraisal results - continued

	AMSTAR 2 Criteria*	Fagan & Catalano (2013)	Fellmeth et al. (2013)	Ferguson & Kilburn (2003)	Fields & McNamara (2003)	Gavine, Donelly, & Williams (2016)	Hahn et al. (2007)	Howard, Flora, & Griffin (1999)	Jiménez-Barbero et al. (2015)	Jouriles et al. (2018)	Katz & Moore (2013)
1	Population, Intervention, Comparison and Outcome	Yes	Yes	No	Uncl	Yes	Yes	Yes	Yes	Yes	Yes
2	Pre-registered protocol	No	Yes	No	No	No	No	No	Partial Yes	No	No
3	Study design criteria	No	No	Uncl	No	No	Yes	Yes	Yes	No	Yes
4	Comprehensive literature search	Partial Yes	Partial Yes	No	Uncl	No	Partial Yes	Partial Yes	Partial Yes	Partial Yes	Uncl
5	Study selection in duplicate	No	Yes	No	Uncl	Uncl	Uncl	No	Yes	No	No
6	Data extraction in duplicate	No	Yes	No	Uncl	Uncl	Yes	No	No	uncl	No
7	List of excluded studies	No	Yes	No	No	No	No	No	No	No	No
8	Detail of included studies	Partial Yes	Partial Yes	No	No	No	No	Partial Yes	No	No	No
9	Risk of Bias (RoB) in individual studies	No	Yes	Uncl	No	Partial Yes	No	Uncl	No	No	No
10	Sources of funding	No	No	No	No	No	No	No	No	No	No
11	Meta-analysis – appropriate statistical methods	Not performed	Yes	No	No	Not performed	Yes	Not performed	Yes	No	Yes
12	Meta-analysis – assess RoB	Not performed	Yes	Uncl	No	Not performed	Yes	Not performed	Yes	No	Yes
13	Meta-analysis – incorporate RoB	No	Yes	No	No	Yes	Yes	Uncl	Yes	No	Yes
14	Explanation for any heterogeneity observed	No	Yes	No	No	Yes	No	No	Yes	No	Yes
15	Publication bias	Not performed	Yes	Yes	No	Not performed	No	Not performed	Yes	No	No
16	Conflicts of interest reported	Yes	Yes	No	No	No	Yes	No	No	Uncl	No

* Critical points are highlighted with yellow

Appendix B. AMSTAR – 2 Quality appraisal results - continued

	AMSTAR 2 Criteria*	Kettrey & Marx (2018)	Lee, Kim, & Kim (2015)	Leen et al. (2013)	Limbos et al. (2007)	Lösel & Beelmann (2003)	Malhotra, Gonzalez-Guarda, & Mitchell (2015)	Nocentini, Zambuto, & Menesini (2016)	Park-Higgerson et al. (2008)	Petering, Wenzel, & Winetrobe (2014)	Polanin, Espelage, & Pigott (2012)
1	Population, Intervention, Comparison and Outcome	Yes	Yes	Yes	Yes	Yes	Yes	Yes	Yes	Yes	Yes
2	Pre-registered protocol	Uncl	No	No	No	No	No	No	No	No	No
3	Study design criteria	Yes	No	No	No	No	No	No	Yes	No	No
4	Comprehensive literature search	Partial Yes	No	No	No	No	Yes	No	No	Partial Yes	No
5	Study selection in duplicate	Yes	Yes	Uncl	No	No	Uncl	Uncl	No	Yes	No
6	Data extraction in duplicate	Yes	Yes	Uncl	No	Yes	Yes	Uncl	Yes	Yes	Yes
7	List of excluded studies	No	No	No	No	No	No	Yes	No	No	No
8	Detail of included studies	Partial Yes	Partial Yes	No	No	No	Partial Yes	Yes	Partial Yes	Partial Yes	No
9	Risk of Bias (RoB) in individual studies	No	No	No	Uncl	No	No	No	No	No	No
10	Sources of funding	No	No	No	No	No	No	No	No	No	No
11	Meta-analysis – appropriate statistical methods	No	Yes	Not performed	Not performed	Yes	Not performed	Not performed	Yes	Not performed	No
12	Meta-analysis – assess RoB	No	No	Not performed	Not performed	No	Not performed	Not performed	No	Not performed	No
13	Meta-analysis – incorporate RoB	No	No	No	No	No	No	No	No	No	No
14	Explanation for any heterogeneity observed	Yes	Yes	Uncl/No	Yes	Yes	No	No	Yes	No	Yes
15	Publication bias	No	No	Not performed	Not performed	Yes	Not performed	Not performed	Yes	Not performed	Yes
16	Conflicts of interest reported	No	No	No	Yes	No	Yes	No	No	No	No

* Critical points are highlighted with yellow

Appendix B. AMSTAR – 2 Quality appraisal results - continued

	AMSTAR 2 Criteria*	Sawyer, Borduin, & Dopp (2015)	Scheckner et al. (2002)	Smith et al. (2004)	Storer, Casey, Herrenkohl (2016)	Ttofi & Farrington (2011)	Vreeman & Carroll (2007)	Whitaker et al. (2006)	Whitaker et al. (2013)	Wilson, Lipsey & Derzon (2003)	Wilson & Lipsey (2007)
1	Inclusion criteria Population, Intervention, Comparison and Outcome	Yes	Yes	No	No	Yes	Uncl	Yes	Yes	Yes	Yes
2	Pre-registered protocol	No	No	No	No	No	No	No	No	No	No
3	Study design criteria	No	Uncl	Yes	No	Yes	Uncl	No	No	No	Yes
4	Comprehensive literature search	Partial Yes	Partial Yes	Partial Yes	No	Partial Yes	Partial Yes	No	Partial Yes	No	No
5	Study selection in duplicate	Yes	No	No	No	No	No	Yes	No	No	No
6	Data extraction in duplicate	Yes	No	Yes	No	No	Yes	Yes	Yes	No	No
7	List of excluded studies	No	No	No	No	No	No	No	No	No	No
8	Detail of included studies	No	Partial Yes	No	Partial Yes	No	Partial Yes	Partial Yes	Partial Yes	No	No
9	Risk of Bias (RoB) in individual studies	No	No	No	No	No	No	No	No	No	No
10	Sources of funding	No	No	No	No	No	No	No	No	No	No
11	Meta-analysis – appropriate statistical methods	Yes	No	Not performed	Not performed	No	Not performed	Not performed	Not performed	No	Yes
12	Meta-analysis – assess RoB	No	No	Not performed	Not performed	No	Not performed	Not performed	Not performed	Yes	No
13	Meta-analysis – incorporate RoB	No	No	No	No	No	No	No	No	No	No
14	Explanation for any heterogeneity observed	Yes	No	No	No	No	No	No	No	No	Yes
15	Publication bias	Yes	No	Not performed	Not performed	No	Not performed	Not performed	Not performed	No	No
16	Conflicts of interest reported	No	No	No	Yes	No	Yes	No	No	No	Yes

* Critical points are highlighted with yellow

Appendix C. Characteristics of the studies

Authors	Type of review	No. of studies	Country	Age	Settings	Behavior outcomes	Bystander component	Quality	Type of violence	Effectiveness for behavior in populations 15-30 years old
Baldry & Farrington (2007)	Meta-analysis	16	Australia Europe Canada USA	8 to 17	School	Perpetration, victimization prevalence rates	Yes, but no reports on effectiveness	Critically Low	Bullying	3 programs demonstrated small effect on reduction of perpetration and victimization, 3 increased perpetration, and 2 increased victimization.
Ferguson & Kilburn (2003)	Meta-analysis	42 (45)	Not specified	School years	School	Nonviolent and violent bullying	No	Critically Low	Bullying	Overall small positive effect but effect sizes too small to be noticeable (including 5 high school studies, age unclear)
Lee, Kim, & Kim (2015)	Meta-analysis	13	Australia China Europe USA	7 to 16	School	Perpetration victimization observations	No	Critically Low	Bullying	Small to moderate positive effects on victimization, including populations 15 years old (4)
Nocentini, Zambuto, & Menesini (2016)	Review	32	Europe USA	School years	School	Perpetration victimization	Yes	Critically Low	Bullying	Positive effect for bullying (2 studies included population of interest)
Polanin, Espelage, & Pigott (2012)	Meta-analysis	11	Europe USA	K to 18 y.o.	School	Bystander helping behaviors	Yes, and reports on effectiveness	Critically Low	Bullying	Small positive effect for bystander behavior across all studies; larger effect sizes for younger students. Shorter programs showed larger effect sizes
Smith et al. (2004)	Review	14	Europe USA	School years	School, parent	Perpetration victimization	No	Critically Low	Bullying	Small negligible effect (3), mixed/harmful effect for perpetration or victimization (2)
Ttofi & Farrington (2011)	Meta-analysis	44 (89)	Europe North America	7 to 18	School	Perpetration victimization	No	Critically Low	Bullying	Evidence of effectiveness for perpetration and victimization for 11 years and older
Vreeman & Carroll (2007)	Review	26 (56)	Australia Europe North America South Africa	7 to 18	School, not limited to	Perpetration victimization	No	Critically Low	Bullying	Harmful effects (1 increased perpetration and victimization), significant reductions in perpetration and victimization (1), no significant changes (1)

Appendix C. Characteristics of the studies (continued)

Authors	Type of review	No. of studies	Country	Age	Settings	Behavior outcomes	Bystander component	Quality	Type of violence	Effectiveness for behavior in populations 15-30 years old
DeKoker et al. (2013)	Review	6 (8)	USA, Canada, South Africa	11 to 26	School, community	Physical and psychological perpetration, victimization, prevalence rates	Yes, but no reports on effectiveness	Critically Low	Dating and relationship	Significant positive effect for perpetration (3), no effect (2)
DeLaRue, Polanin, Espelage, & Piggott (2017)	Meta-analysis	23	USA, Canada	11 to 18	School	Perpetration victimization	Yes, but limited data	High	Dating and relationship	Small nonsignificant effect on perpetration (2) and small significant effect on victimization (unclear/none for 15 and older) was found, however, effect sizes decreased to null at follow-up.
Edwards and Hinsz (2014)	Meta-analysis	8	Not specified	School years	School	Self-reported behaviors, prevalence rates	No	Critically Low	Dating and relationship	Small positive effect (overall, not specified for behaviors), interestingly, small harmful effect on attitudes (2)
Fellmeth et al. (2013)	Meta-analysis	38	USA South Korea	12 to 25	School, university	Perpetration, victimization, bystander, prevalence rates	Yes, but no reports on effectiveness	Moderate	Dating and relationship	No statistically significant effect
Leen et al. (2013)	Review	9	Europe North America	12 to 18	School, not limited to	Perpetration, victimization, negative dating behavior, substance, condom use	No	Critically Low	Dating and relationship	Small positive effect for negative dating behavior (1)
Malhotra, Gonzalez-Guarda, & Mitchell (2015)	Review	18 (22)	Canada, USA, not specified	School years	School, family, community	Perpetration victimization	Yes	Critically Low	Dating and relationship	Positive effect decreased over time in several programs; harmful effect or perpetration (1); positive effect maintained at a 4-year follow-up (1); positive effect on victimization but not perpetration (1)

Appendix C. Characteristics of the studies (continued)

Authors	Type of review	No. of studies	Country	Age	Settings	Behavior outcomes	Bystander component	Quality	Type of violence	Effectiveness for behavior in populations 15-30 years old
Petering, Wenzel, & Winetrobe (2014)	Review	14	USA, Canada	12 to 26	School, not limited to	Victimization Perpetration, bystander behavior, condom use	Yes	Critically Low	Dating and relationship	Positive effects in 3 programs for perpetration and victimization, condom use (1); 1 program effective for bystander behavior in athletes
Storer, Casey, Herrenkohl (2016)	Review	9 (15)	USA, not specified	College years	College	Bystander behavior	Yes	Critically Low	Dating and relationship	Small but significant effect on bystander behavior (4); no effect (2); mixed effect (3). Perpetration or victimization not measured.
Whitaker et al. (2006)	Review	11	Not specified	School years	School community	Perpetration Victimization Psychological perpetration	No	Critically Low	Dating and relationship	Small improvement for perpetration and victimization in 1 study, including a 4-year follow-up (up to 14 years old); small decreases in physical perpetration in 1 program (effect larger for girls); 2 studies reported no or negligible effect for behavior.
Whitaker et al. (2013)	Review	19	South Africa, USA, not specified,	School years	School and other settings	Perpetration Victimization	No	Critically Low	Dating and relationship	Small improvement for perpetration and victimization in 1 study, including a 4-year follow-up (population up to 14 years old)
Anderson & Whiston (2005)	Meta-analysis	102 (69)	USA	Mean age 20	School	Perpetration Victimization	No	Critically Low	Sexual assault	No significant effect on behavior
DeGue et al. (2014)	Review	140	Not specified	10 to 47	School, college	Perpetration victimization, bystander behavior	Yes, and reports on effectiveness	Critically Low	Sexual assault	Positive effect for perpetration and victimization (1); positive effects on perpetration (1); mixed results for bystander behavior (1, unclear); harmful effect for college men (1)
Jouriles et al. (2018)	Review	24	Not specified	Underg raduate	College	Bystander behavior	Yes, and reports on effectiveness	Critically Low	Sexual assault	Small significant effect for bystander behavior that decreased at follow-up; perpetration and victimization not reported

Appendix C. Characteristics of the studies (continued)

Authors	Type of review	No. of studies	Country	Age	Settings	Behavior outcomes	Bystander component	Quality	Type of violence	Effectiveness for behavior in populations 15-30 years old
Katz & Moore (2013)	Meta-analysis	12	Not specified	Avg 19	College	Perpetration, bystander behavior	Yes, and reports on effectiveness	Critically Low	Sexual assault	Moderate effects for bystander behavior, no effects for perpetration
Kettrey & Marx (2018)	Meta-analysis	15	Not specified	College	College	Bystander behavior	Yes, and reports on effectiveness	Critically Low	Sexual assault	Small significant positive effect for bystander behavior; perpetration and victimization not reported
Derzon (2006)	Meta-analysis	83	Not specified	5 to 18	School	Aggression, perpetration, criminal behavior, prevalence rates	No	Critically Low	Antisocial	Positive effects for antisocial and aggressive behavior, however, it was unclear for which age groups
Fields & McNamara (2003)	Meta-analysis	Uncl.	Not specified	Youth, not specifie d	School community facility	Aggression, perpetration and victimization, prevalence rates	No	Critically Low	Antisocial	Significant positive effects in primary 3 studies compared to control groups (however outcome measures, as well as populations were unclear)
Gavine, Donelly, & Williams (2016)	Review	16 (21)	Not specified	11 to 18	School	Aggression, perpetration victimization, prevalence rates	No	Critically Low	Antisocial	Significant positive effect on violence rates, and victimization; significantly lesser increase in violence rates(1); no effect (1)
Lösel & Beelmann (2003)	Meta-analysis	84	Australia Canada Europe USA	4 to 18	School, not limited to	Perpetration Victimization	No	Critically Low	Antisocial	Small significant positive effect overall across studies
Park-Higgerson et al. (2008)	Meta-analysis	26	USA	1st Grade to 17 y.o.	School	Perpetration Victimization	No	Critically Low	Antisocial	Moderate effect for antisocial behavior that remained at a 4-year follow-up; however, it was unclear how many preventive programs were school-based and focused on our target age

Appendix C. Characteristics of the studies (continued)

Authors	Type of review	No. of studies	Country	Age	Settings	Behavior outcomes	Bystander component	Quality	Type of violence	Effectiveness for behavior in populations 15-30 years old
Sawyer, Borduin, & Dopp (2015)	Meta-analysis	66	Not specified	Birth to 17 y.o.	School community parent	Perpetration Victimization	No	Critically Low	Antisocial	Moderate effect for antisocial behavior that remained at a 4-year follow-up; however, it was unclear how many preventive programs were school-based and focused on our target age
Wilson, Lipsey & Derzon (2003)	Meta-analysis	221 (362)	USA, not specified	School years	School	Perpetration Victimization	No	Critically Low	Antisocial	Small positive effect for antisocial behavior overall; larger effects in populations with higher prevalence of violence, including high school sample
Wilson & Lipsey (2007)	Meta-analysis	249	Australia, Canada, UK, USA	School years	School	Perpetration Victimization	No	Critically Low	Antisocial	Small positive effect for antisocial behavior overall, including high school sample (those 14 years and older).
Atienzo, Baxter, & Kaltenthaler (2017)	Review	10 (9)	Latin America	10 to 24	School, favela, gang, community	Perpetration, witnessed violence, prevalence rates	No	Low	General: bullying, antisocial behavior	Small reductions in witnessed bullying (1), small reductions in perpetration and witnessing antisocial behaviors among peers (1), increased involvement in deviant activities (1)
Cassidy, Bowman, McGrath, & Matzopoulos (2016)	Review	6	Europe and USA	10 to 29	School, university	Perpetration Victimization	No	Critically Low	General: antisocial, sexual assault	Small positive effect in 6 programs for populations including 15 years and older, perpetration and victimization
Cox et al. (2016)	Review	17 (19)	Australia	12 to 18	School, community	Perpetration Victimization	No	Critically Low	General: bullying, substance abuse related, antisocial	Antisocial - significant positive effect for physical violence (1); substance abuse: no report of effectiveness for age >15; bullying: small reductions in small sample (1)

Appendix C. Characteristics of the studies (continued)

Authors	Type of review	No. of studies	Country	Age	Settings	Behavior outcomes	Bystander component	Quality	Type of violence	Effectiveness for behavior in populations 15-30 years old
Fagan & Catalano (2013)	Review	17	Not specified	School years	School community family other	Perpetration, violence rates, fighting rates	No	Critically Low	General: antisocial relationship substance abuse	Small to moderate positive effects for dating violence and bullying (2), with long term effects up to 3 years; substance abuse: no report of effectiveness for age 15
Hahn et al. (2007)	Meta-analysis	53	High-income countries	Student s	School, other	Perpetration, victimization, substance abuse-related harms	No	Critically Low	General: bullying relationship substance antisocial	Small positive effect at all levels for substance abuse-related harms, bullying, relationship violence, and antisocial behavior, but not maintained at follow-up. Larger effect sizes for dating and antisocial behavior
Howard, Flora, & Griffin (1999)	Review	44	US	School years	School	Aggression, shy behavior, perpetration victimization, fight-related nurse visits, suspension rates	No	Critically Low	General: bullying, relationship antisocial	Bullying - small positive effect, no effect compared with control, effect only for boys (2); dating violence: only changes for attitudes measured (1)
Jiménez-Barbero et al. (2015)	Meta-analysis	14	Australia EuropeU SA	7 to 16	School	Aggression, perpetration Victimization substance abuse-related harms, frequency	No	Critically Low	General: bullying, substance, antisocial	Small positive effect for substance abuse-related harms in 2 programs
Limbos et al. (2007)	Review	41	Not specified	12 to 17	School, community facility	Perpetration, substance abuse related harms	No	Critically Low	General: antisocial relationship substance abuse	Not effectivene for ages 15 and older
Scheckner et al. (2002)	Meta-analysis	16	Not specified	School years	School, community parent	Perpetration and victimization	No	Critically Low	General: antisocial substance abuse	No effectiveness (negligible results) in 2 studies
