# Supplementary material for: What Works in Violence Prevention Among Young People?: A Systematic Review of Reviews
Source: Trauma Violence Abuse. 2020 Jul 17;23(5):1388–404. doi: 10.1177/1524838020939130 (PMC9606003; doi:10.1177/1524838020939130)
Supplement: Supplemental Material, 2602_Key_findings_and_implications - What Works in Violence Prevention Among Young People?: A Systematic Review of Reviews [file 2602_Key_findings_and_implications.pdf]

Table 2. Critical findings

• Reviews and meta-analyses demonstrated significant improvements in knowledge and attitudes.<br>• Not all programs evaluated behavior as an outcome measure, and the findings for behavior change were mixed.<br>• Small but significant reductions in perpetration and victimization were reported but effects tended to fade or diappear at follow up in the majority of programs.<br>• The lack of rigorous longitudinal evaluation design and moderator analyses limited our ability to draw conclusions about specific program features that enhance effectiveness of violence prevention programs.<br>• We provide a set of recommendations identifying best bet content and guidelines on how to improve evaluation methodology in this field.

Table 3. Implications for practice, policy and research

• A moderator analysis should be carried out to explore which program features enhance or decrease effectiveness of programs depending on sample and context.<br>• Future programs should incorporate a gender-neutral approach and be implemented in various settings to ensure inclusivity and diversity of populations.<br>• Successful campus-based violence prevention programs should include behavior change in its various forms, such as physical, verbal, and emotional, as the target outcome.<br>• Program curricula should be intensive and activity-based with a skill-building component.<br>• A needs assessment should be performed to ensure the timely and appropriate implementation.<br>• If future programs incorporate our evidence-based recommendations, they may tackle violence among young people more effectively.<br>• When more persuasive effectiveness data is generated the most effective approaches can be integrated into nationally-funded programs embedded in everyday education practice
